# Endoscopic Management of Benign Gallbladder Diseases

**DOI:** 10.5152/tjg.2024.24149

**Published:** 2024-09-01

**Authors:** Yimei Ji, Weirong Huang, Jiefang Guo, Bing Hu

**Affiliations:** 1 Department of Gastroenterology and Endoscopy, Eastern Hepatobiliary Surgery Hospital, Naval Medical University, Shanghai, China

**Keywords:** GBDs, endoscopic ultrasound, endoscopic retrograde cholangiopancreatography, natural orifice transluminal endoscopic surgery, management

## Abstract

Acute cholecystitis, cholelithiasis, and gallbladder polyps represent the most gallbladder benign diseases. Endoscopic approaches for the management of these diseases were an alternative to standard laparoscopic cholecystectomy. These endoscopic approaches include transpapillary approaches via endoscopic retrograde cholangiopancreatography, transmural access approaches via endoscopic ultrasound, and endoscopic surgical approaches using natural orifice transluminal endoscopic surgery approaches. However, it’s still uncertain which approach is associated with the superior clinical outcomes due to the lack of high-level evidence. Our review provides new insight into the endoscopic approaches for the management of gallbladder benign diseases, with the latest evidence included.

**Main Points**

- Endoscopic approaches for the management of benign gallbladder benign diseases (GBDs) were an alternative to standard laparoscopic cholecystectomy.
- It is still uncertain which approach is associated with the best overall long-term clinical outcomes due to the lack of high-level evidence.
- The current review provides new insight into the endoscopic approaches for the management of GBDs, with the latest evidence included.

## Introduction

Gallbladder benign diseases (GBDs) are common and annually impact a sizable portion of the population. It is estimated that gallbladder disease affects an estimated 20 million Americans.^1^ Although cholelithiasis, gallbladder polyps, and chronic cholecystitis are additional manifestations of GBD, acute cholecystitis is by far the most frequent form of the illness.

The gold standard for treatment of GBD has always been surgery, namely cholecystectomy. In cases where surgery is neither appropriate nor practical, percutaneous gallbladder treatment is a commonly recognized, efficient, and accessible therapeutic approach.^2^ However, percutaneous procedures force patients to use a heavy external drain that needs to be changed frequently, increasing the patient’s risk of infection, developing a permanent fistula, and having a lower quality of life.^3,4^ When the percutaneous approach is not recommended due to coagulopathy or ascites in poor surgical candidates, endoscopic methods can be an alternative approach.^5^ There are now a number of endoscopic techniques available as alternatives for percutaneous or surgical procedures. These techniques include transpapillary approaches via Endoscopic Retrograde Cholangiopancreatography (ERCP), transmural access approaches via endoscopic ultrasound (EUS), and endoscopic surgical approaches using natural orifice transluminal endoscopic surgery (NOTES) approaches. Our review focuses on clinical outcomes of endoscopic approaches to the management of GBD.

## ERCP-guided Transpapillary Approach

### Gallbladder Drainage

ERCP-guided gallbladder intervention was first described in 1990. The primary goal of this technique was to drain the gallbladder.^6^ Following the standard bile duct cannulation, an obliquely angled guidewire is advanced to the cystic duct and into the gallbladder, where it is coiled. After that, 2 drainage methods were described: nasogallbladder drainage (NGBD) and transpapillary cystic duct stent (TPCDS) placement. NGBD allows for continued flushing and irrigating of the gallbladder. TPCDS allows for the removal of residual cystic duct stones. A study reported that the technical, clinical success, and incidence of complications in ERCP-guided NGBD and TPCDS were 81%, 75%, and 3.6%, respectively, versus 96%, 88%, and 6.3%.^7^ The authors concluded that ERCP-guided gallbladder drainage was a good alternative drainage method for acute cholecystitis.

Other studies also reported the clinical outcomes of ERCP-guided drainage of the gallbladder. In 2015, a study by McCarthy et al concluded that ERCP-guided TPCDS was successfully performed in 22 patients (76%), with 18 being successful on the first attempt. During a mean follow-up of 12 months, the clinical response rate was 90% after stent placement. Only two patients developed delayed complications.^8^ Another Japanese retrospective study reported that the technical success rate was 77.5% for elderly patients, and no immediate postprocedural complications occurred. The majority of patients (93.5%) remained asymptomatic until they died or the end of the research period, and the recurrence rate of cholecystitis was as low as 3.3%.^9^ The complication rates were higher in a prospective study compared with the above retrospective studies. With 29 patients included, the immediate adverse events were mild pancreatitis (8.7%) and cholestasis (8.7%). During the follow-up period (median 586 days, range 11-1403 days), late adverse events developed in 20% of patients. The technical success rate was 79.3% in their study.

We noticed that the technical success rate was less than 80% in all the relevant studies, which was significantly lower than standard ERCP-guided bile duct intervention.^10^ Most likely, this is because traditional fluoroscopic guiding makes it impossible to enter the cystic duct. Peroral cholangioscopy can facilitate NGBD and cystic duct stent placement by providing better direct vision of the cystic duct.^11-15^ Ridtitid et al^16^ reported that in 103 poor surgery candidates, only 55 (53%) patients had successful ERCP-guided TPCDS. The overall technical success rate of ERCP-guided TPCDS increased by 22% after additional peroral cholangioscopy assistance without additional complications. Similarly, Yoshida et al^17^ concluded that peroral cholangioscopy-guided gallbladder drainage achieved a significantly higher success rate than conventional ERCP-guided gallbladder drainage alone (94.1% vs. 73.2%, *P* = .003). To further improve the cystic duct cannulation success rate, a newly designed peroral cholangioscopy with an ultrafine outer diameter that facilitates gallbladder visualization was reported by Zhou et al.^18^ To further improve the cystic duct cannulation success rate, a newly designed peroral cholangioscopy with an ultrafine outer diameter that facilitates gallbladder visualization was reported by Zhou et al. ^18-20^

### Gallstones

ERCP-guided gallbladder intervention also allows for the treatment of gallstones, especially in patients with Mirizzi syndrome, using peroral cholangioscopy.^21,22^ The clinical outcomes were satisfactory. Tsuyuguchi et al^23^ reported that 47 patients had type II Mirizzi Syndrome and they were all successfully treated by peroral cholangioscopy-directed lithotripsy. Only 4 patients experienced stone recurrence. Bhandari et al^24^ performed single-operator cholangioscopy-guided laser lithotripsy in 34 patients with cystic duct stones and Mirizzi syndrome. In 32 individuals (94%), ductal clearance was obtained in a single session. All the adverse events were mild.^24^ In general, peroral cholangioscopy-directed lithotripsy may be a good approach for the treatment of cystic duct stones and Mirizzi syndrome. Large-scale prospective studies are still needed to verify the clinical outcomes of the technique.

There were few data reported about the ERCP-guided gallbladder intervention for the treatment of gallstones in the gallbladder. Liu et al described a pregnant woman who suffered from acute cholecystitis. After being squeezed into the cystic duct, using the clear cap as guidance, the cholangioscope extended and straightened the curved cystic duct to enable a smooth penetration into the gallbladder. Under cholangioscopy, the gallstones were extracted using a narrow basket and moved to the common bile duct. Using a standard ERCP basket, the common bile duct stones and gallbladder origin stones were removed after the papillary orifice was cut and expanded.^15^ Due to the technical difficulty, the above procedure may not be a good alternative approach to treat gallstones in the gallbladder.

### Gallbladder Polypoid Lesions

Similarly, due to the technical difficulty, ERCP-guided gallbladder intervention is rarely performed for the treatment and diagnosis of gallbladder polypoid lesions. Kamada et al^25^ reported that an 85-year-old woman presented with acute cholangitis related to CBD stones. Preoperative contrast-enhanced computed tomography (CT) identified a suspicious 20 mm gallbladder tumor with enhancement. A nodular, elevated lesion was identified by cholangioscopy at the gallbladder fundus. However, the biopsy was not conducted because the forceps could not pass through the curved cholangioscope.^25^ The novel ultrafine peroral cholangioscopy may also help visualize the internal gallbladder.^18^ However, until more flexible instruments are used in clinical practice, the ERCP-guided gallbladder intervention will remain technically difficult (Table 1).

## EUS-guided Transmural Access Approaches

### Gallbladder Drainage

When treating high-surgical-risk patients with acute calculous and acalculous cholecystitis, EUS-guided gallbladder drainage (EUS-GBD) with the placement of a lumen-apposing metal stent (LAMS) ought to be the primary option rather than cholecystectomy in institutions with sufficient volumes and expertise.^26^ In the past, nasogallbladder tube,^27,28^ plastic stents,^29^ and self-expanding metal stents^30,31^ were used to perform gallbladder drainage. However, due to the high risk of bile leak, peritonitis, stent migration, pneumoperitoneum, and limited drainage efficacy, EUS-guided gallbladder was not widely applied in clinical practice.^32^ The most significant change in EUS-guided gallbladder intervention has been the development of the LAMS. Depending on which provides better EUS imaging windows and the endoscopist’s skill, the stent may be implanted via a transgastric or transduodenal approach. Moreover, the sinus tract produced by LAMS facilitates the clearance of gallstones before removing the stent.^33^

Many new types of LAMS have been applied in gallbladder drainage. In 2014, Teoh et al^34^ recommended that EUS-GBD be carried out using a LAMS device that has integrated electrocautery, enabling stent placement under EUS supervision with just one instrument. In 2022, a novel electrocautery-enhanced LAMS was reported to be feasible in interventional EUS procedures, but the data on gallbladder drainage was scarce.^35^ In 2023, Brandaleone et al^36^ described a new dedicated electrocautery LAMS for gallbladder drainage. The technical success of LAMS placement was 100%, and clinical success was 76.67%. Adverse events were observed in 2 patients (5.6%). However, comparison studies to determine which type of LAMS are still scarce.

The treatment outcomes for EUS-GBD were favorable. A meta-analysis with 104 patients who had malignant biliary obstruction concluded that the pooled rates of clinical success were 85% with significantly decreased preprocedure bilirubin.^37^ Another meta-analysis with 477 patients included reported that more than 90% of patients who underwent EUS-GBD achieved technical and clinical success.^38^ An earlier meta-analysis with 1004 patients reported that the pooled technical success and clinical success both reached over 95%.^39^

The reported post-procedure complication rates were varied but occurred infrequently. In 2018, a meta-analysis summarized that complications included stent dislodgement, stent blockage, leakage, peritonitis, pneumoperitoneum, abdominal abscess, and recurrent cholecystitis. These morbidities were observed in 20.4% of patients. The primary cause of the 3.9% overall death rate was persistent sepsis.^40^ The morbidity rate decreased to 14.8% in an updated 2022 meta-analysis. Procedure-related mortality was 0.1%.^39^ Technical success rate and the overall clinical success rose when center experience was proxied to more than 10 instances per year. Another recent meta-analysis concluded that compared to other modalities, EUS-GBD exhibited the lowest risk of recurrent cholecystitis.^41^ Therefore, EUS-GBD remains a safe procedure in expert centers. 

Following the EUS-GBD cholecystitis resolution, there were 2 stent management options. One option is to follow up for 4-6 weeks after initial drainage. A repeat endoscopic examination is performed to see if gallstones have passed spontaneously. Successfully performed in 93.1% of patients, the double pigtail plastic stents were replaced with LAMS to maintain the fistula.^42^ More than half of gallstones will pass spontaneously through the sinus tract.^42^ Patients who are too weak or who refuse a second treatment are frequently given the second alternative, which is to leave the LAMS in place permanently. This is a feasible strategy for some very high-risk patients, as evidenced by research detailing the long-term results (median follow-up duration, 275 days) of EUS-GBD, which showed minimal rates of delayed adverse events (7.1% and 86% stent patency after 3 years). ^43^

### Gallstones and Other Non-Cholecystitis Gallbladder Diseases

As mentioned above, the sinus tract produced by LAMS facilitates the clearance of gallstones. However, limited studies have reported the treatment outcomes of EUS-GBD using LAMS for gallstone removal. The study by Chan et al reported that 88% of stones were removed after a mean of 1.25 endoscopy sessions.^42^ In another study from China, seven patients with symptomatic cholelithiasis received transgastric or transduodenal EUS-guided LAMS placement. The patients had a repeat endoscopic operations for cholecystolithotomy with basket stone removal 2 weeks after LAMS installation. During a follow-up period of 3 to 20 months, all 7 patients experienced minor side effects and no recurrence of cholelithiasis following their procedures, which was effective.^44^ Fifteen patients who received EUS-GBD for non-cholecystitis causes are listed in another retrospective analysis. After EUS-GBD treatment, the symptoms of all but one of the reported patients were resolved, and a 13.3% risk of recurrent symptomatic biliary illness was seen after a year of follow-up.^44^ Overall, more research is required in this area, but endoscopic management of benign gallbladder diseases other than cholecystitis seems to be a practical and viable option for patients seeking the least invasive course of treatment and/or gallbladder preservation.

### Gallbladder Polypoid Lesions

The surgical indications for gallbladder polyps were greater than or equal to 10 mm in diameter.^45^ It has been shown that EUS is more accurate than conventional transabdominal ultrasonography in distinguishing gallbladder polyps from gallbladder pseudo-polyps.^46,47^ The advantage of EUS may be that it helps prevent unnecessary surgeries. Moreover, EUS-guided tissue acquisition helps distinguish benign and malignant gallbladder polyps with a high yield (90.9%).^48,49^ The data obtained from EUS and EUS-guided tissue collection helped with the patients’ ongoing clinical care.

The next step in the treatment of gallbladder polyps would be endoscopic excision following LAMS placement, as access to the gallbladder is now a reasonably simple procedure with LAMS. In a small case study from China, a gastroscope was pushed into the gallbladder for polyp resection a few days after the LAMS was implanted. At the end of the procedure, hemostatic clips were used to close the fistula. Technically, all patients had a successful operation, and only one patient later had a cholecystectomy because of a cholelithiasis recurrence.^50^ The results of this case series indicated that EUS-guided gallbladder polyp removal after LAMS placement may be an effective therapeutic option for small gallbladder polyps, saving the patient from cholecystectomy and lowering the requirement for follow-up ultrasonographic assessments. With reference to this interventional method, more research is required (Table 2).

## Natural Orifice Transluminal Endoscopic Surgery

For those who were not suitable or unwilling to undergo laparoscopic cholecystectomy, NOTES cholecystolithotomy with or without gallbladder excision may be an alternative approach. NOTES involves inserting a flexible endoscope via a natural channel into the human body. This technique may cause less discomfort as it prevents cutaneous scarring by not extracting the surgical sample via the skin.

### Cholecystectomy

There were several approaches obtained for endoscopists to access the peritoneal cavity: transgastric, transrectal, or transvaginal approaches. Tsin et al reported the first NOTES cholecystectomy in 2003 through the transvaginal approach.^51^ Since then, several studies have reported that NOTES cholecystectomy was associated with better aesthetic outcomes, shorter procedure duration, shorter hospital stay, and less pain compared with the standard laparoscopic approach.^52-56^ However, 2 randomized controlled studies did not reveal any differences in adverse events, hospital stays, or postoperative discomfort between NOTES and laparoscopic approaches, but NOTES was associated with a longer procedure duration.^57,58^ A meta-analysis also concluded that there was no significant difference between the safety of NOTES and laparoscopic cholecystectomy. NOTES is associated with a higher rate of intraoperative conversion when compared with laparoscopic cholecystectomy.^59^ It is worth mentioning that no cases of sexual dysfunction have been reported after the transvaginal approach was applied.^60^ The studies to determine which access approach is superior are still lacking.

Easy access to the gallbladder and early recovery are benefits of transrectal NOTES. Moreover, the supine position of the patient facilitates a thorough cleaning of the peritoneal cavity. The necessity for intestinal preparation and cleanliness prior to the surgery was a drawback.^61^ Conversely, the advantage of transgastric NOTES is that no preoperative bowel cleansing is required. The following are disadvantages: it is more challenging to reach the gallbladder, make the incision, and remove the stones. There is a delay in fluid intake, and a drainage tube might be needed. There is trouble keeping the endoscope steady. Due to the patient’s left lateral posture, it may be more difficult to thoroughly clean in the event of bile leakage, which could raise the risk of peritonitis. Additionally, because the stomach stoma has a thicker wall than the rectal stoma, closing it is more challenging.^61^ The transvaginal approach had the closest pathway to the gallbladder. The advantages and disadvantages were similar to those of the transrectal approach. Obviously, this approach is specific to women.

In general, NOTES is still in its early stages of development because the anticipated technological challenges brought on by the lack of specialized NOTES scopes and accessories significantly restrict its broad adoption and distribution. The endoscopists were not familiar with the anatomy of the abdominal cavity, and the surgeons were not familiar with the operation of endoscopy, which may significantly influence the clinical outcomes of NOTES.^32^

### Gallbladder-Preserving Cholecystolithotomy

The gallbladder-preserving cholecystolithotomy through NOTES is less performed than cholecystectomy due to the risk of bile leak and the lack of a proper instrument. In 2015, Liu et al^62^ first reported a successful trans-rectal NOTES procedure for gallbladder-preserving cholecystolithotomy. In 2020, Li and Han^63^ first reported the transgastric NOTES procedure for gallbladder-preserving surgery. In 2022, Zhang et al^64^ reported the outcomes of the transgastric NOTES procedure, with 22 patients included. The procedures were successfully performed on all patients. The median time for NOTES was 118 minutes. During hospitalization, 4 patients suffered localized peritonitis (4/22, 18.2%), and no other complications occurred. After receiving conservative medical treatment, all these patients achieved a full recovery. After a median follow-up of 4 months, only 1 patient suffered residual gallstones.^64^ The propensity-matched study by Ullah et al with 110 patients who underwent NOTES concluded that NOTES was associated with shorter post-procedure fasting and less incidence of post-procedure diarrhea. The gallstone recurrence rate was significantly higher in the NOTES group (10.5%).^65^

Overall, NOTES cholecystolithotomy is a relatively novel treatment, with only a small number of centers having experience with it. There are still few long-term follow-up studies available on gallbladder dysfunction, cholecystitis, abdominal adhesions, and stone recurrence. As stated by Ullah et al,^65^ the development of a dependable technique to stop gallstones from recurring may be necessary given the extensive usage of NOTES cholecystolithotomy (Table 3).

## Recommendations Based on Authors’ Experience

Despite the numerous endoscopic approaches that have been described to manage GBD, laparoscopic cholecystectomy should be the first choice under any circumstance due to the proven safety and efficacy of the technique. Patients who are unwilling or unable to undergo laparoscopic cholecystectomy should be sent to an experienced endoscopic center for further endoscopic treatment. In other clinical conditions, such as malignant distal biliary obstruction, gallbladder drainage can be a rescue therapy after ERCP failure.

Among all the endoscopic procedures, EUS should be the first choice due to the development of LAMS. The large fistula produced by LAMS not only facilitates the transmural drainage of the gallbladder but also provides access, which aids in the therapy of other non-cholecystitis gallbladder diseases. ERCP-guided gallbladder intervention can be considered when peroral cholangioscopy is available, especially for patients with Mirizzi syndrome. For other benign diseases besides acute cholecystitis, ERCP-guided gallbladder intervention should not be the first choice due to the limitations of the instrument and the high incidence of complications after ERCP. NOTES should be performed in stable patients, particularly those who fear body scars. However, the endoscopist may not be familiar with the anatomy and structure of the abdominal cavity, leading to prolonged surgery time and uncontrolled complications.

In general, the indications for endoscopic treatment of gallbladder benign disease are not fully clarified yet. The endoscopist should perform these procedures according to the available instruments and their expertise. Guidelines or an expert consensus are needed to help us better perform these procedures.

## Conclusion

Gallbladder benign disease is one of the most common gastrointestinal conditions and can be associated with significant health threats. For patients who are not deemed appropriate or are unwilling to undergo surgery, alternative approaches are needed. Endoscopic management approaches, including ERCP-guided transpapillary gallbladder intervention, EUS-guided transmural gallbladder intervention, and NOTES, have the advantage of providing comfort and physiologic interventions. However, it’s still uncertain which approach provides the safest, most effective, and best overall long-term clinical outcomes due to the lack of prospective, long-term, large sample size and comparative data. Our review provides new insight into the endoscopic approaches for the management of GBD. The clinical outcomes, advantages, and disadvantages of each approach, with the latest evidence, were presented in our review. We hope our review can help endoscopists understand the field better.

## Figures and Tables

**Table 1.** Summary of Main Research Articles of ERCP-Guided Transpapillary Approach for the Treatment of Gallbladder Benign Diseases

Author	Publication Year	Indication	n	Technical Success Rate, %	Clinical Success Rate, %	Adverse Event Rates, %
Itoi et al^7^	2010	Gallbladder drainage	NSGBD: 194 Stent: 127	81 and 96	75 and 88	3.6 and 6.3
McCarthy et al^8^	2015	Gallbladder drainage	29	76	90	6.9
Maekawa et al^9^	2013	Gallbladder drainage	46	77.5	93.5	0
Ridtitid et al^16^	2020	Gallbladder drainage	Fluoroscopic guidance: 104 SOC guidance: 41	53 and 56	100	11 and 2
Yoshida et al^17^	2021	Gallbladder drainage	Fluoroscopic guidance: 101 SOC guidance: 13	72.3 and 84.6	NR	10.9 and 7.7
Zhou et al^18^	2023	Gallbladder drainage	16	87.5	NR	6.3
Tsuyuguchi et al^23^	2011	Stone removal	43 with Mirizzi syndrome	95.9	90.2	NR
Bhandari et al^24^	2016	Stone removal	34	94	NR	17.6

* A systematic review and 12 cases of EUS-guided gallbladder drainage were included; # single-operator peroral cholangioscopy (SOC) was performed after the initial fluoroscopic guidance procedure was failed; & using a newly designed SOC with an ultrafine outer diameter.

NR: not reported.

**Table 2.** The Summary of Main Research Articles of EUS -guided Transmural Access Approaches for the Treatment of Benign Gallbladder Diseases

Author	Publication Year	Indication	Stent Type	n	Technical Success Rate, %	Clinical Success Rate, %	Adverse Event Rates, %
Penas-Herrero et al^27^	2015	Gallbladder drainage	All types	155	97.5	99.3	8.0
Anderloni et al^28^	2016	Gallbladder drainage	All types	166	95.8	93.4	12.0
Song et al^29^	2010	Gallbladder drainage	plastic	8	100	100	37.5
Kamata et al^30^	2017	Gallbladder drainage	SEMS	12	100	100	0
Teoh et al^34^	2021	Gallbladder drainage	LAMS	30	100	93.3	13.3
Kamal et al^37^	2023	Gallbladder drainage	LAMS and SEMS	104	NR	85	13
Boregowda et al^38^	2023	Gallbladder drainage	All types	477	89.9	97	14.6
Fabbri et al^39^	2022	Gallbladder drainage	All types	1004	98.0	95.4	14.8
Chan et al^42^	2017	Stone removal	LAMS	25	93.1	88.0	0
Choi et al^43^	2014	Gallbladder drainage	SEMS	63	98.4	98.4	11.9
Shen et al^50^	2020	Gallbladder polyp resection	LAMS	6	100	100	16.7

* Systematic review.

SEMS, self-expandable metal stent; LAMS, lumen apposing metal stent; NR, not reported.

**Table 3.** The Summary of Main Research Articles of Natural Orifice Transluminal Endoscopic Surgery (NOTES) for the Treatment of Benign Gallbladder Diseases

Author	Publication year	Indication	Routine	n	Technical success rate, %	Clinical success rate, %	Adverse event rates, %
Dhillon et al^52^	2017	Cholecystectomy	Transvaginal	257	99.2	100	0
Zornig et al^53^	2011	Cholecystectomy	Transvaginal	100	100	100	0
Zornig et al^54^	2008	Cholecystectomy	Transvaginal	20	100	100	0
Bulian et al^55^	2015	Cholecystectomy	Transvaginal	20	100	100	10
Brescia et al^56^	2013	Cholecystectomy	Transvaginal	21	100	100	0
Borchert et al^58^	2012	Cholecystectomy	Transvaginal	20	95	100	10
Zhang et al^64^	2022	Gallbladder-preserving surgery	Transgastric	22	100	100	18.2
Ullah et al^65^	2022	Cholecystolithotomy	Transgastric	86	98.9	100	0

## References

[1] Gallaher JR Charles A . Acute cholecystitis: a review. JAMA. 2022;327(10):965 975. (10.1001/jama.2022.2350) 35258527

[2] Yamashita Y Takada T Kawarada Y , et al. Surgical treatment of patients with acute cholecystitis: Tokyo Guidelines. J Hepatobil Pancreat Surg. 2007;14(1):91 97. (10.1007/s00534-006-1161-x) PMC2784499 17252302

[3] de Mestral C Gomez D Haas B Zagorski B Rotstein OD Nathens AB . Cholecystostomy: a bridge to hospital discharge but not delayed cholecystectomy. J Trauma Acute Care Surg. 2013;74(1):175 180. (10.1097/TA.0b013e31827890e1) 23271093

[4] Saumoy M Yang J Bhatt A , et al. Endoscopic therapies for gallbladder drainage. Gastrointest Endosc. 2021;94(4):671 684. (10.1016/j.gie.2021.05.031) 34344541

[5] Venara A Carretier V Lebigot J Lermite E . Technique and indications of percutaneous cholecystostomy in the management of cholecystitis in 2014. J Visc Surg. 2014;151(6):435 439. (10.1016/j.jviscsurg.2014.06.003) 25168577

[6] Feretis CB Manouras AJ Apostolidis NS Golematis BC . Endoscopic transpapillary drainage of gallbladder empyema. Gastrointest Endosc. 1990;36(5):523 525. (10.1016/s0016-5107(90)71134-0) 2227333

[7] Itoi T Coelho-Prabhu N Baron TH . Endoscopic gallbladder drainage for management of acute cholecystitis. Gastrointest Endosc. 2010;71(6):1038 1045. (10.1016/j.gie.2010.01.026) 20438890

[8] McCarthy ST Tujios S Fontana RJ , et al. Endoscopic transpapillary gallbladder stent placement is safe and effective in high-risk patients without cirrhosis. Dig Dis Sci. 2015;60(8):2516 2522. (10.1007/s10620-014-3371-4) 25287001

[9] Maekawa S Nomura R Murase T Ann Y Oeholm M Harada M . Endoscopic gallbladder stenting for acute cholecystitis: a retrospective study of 46 elderly patients aged 65 years or older. BMC Gastroenterol. 2013;13:65. (10.1186/1471-230X-13-65) 23586815 PMC3675408

[10] DA-Silva RRR Mafra LGA Brunaldi VO Almeida LFD Artifon ELA . Endoscopic ultrasound-guided biliary drainage: a literature review. Rev Col Bras Cir. 2023;50:e20233414. (10.1590/0100-6991e-20233414-en) 36995833 PMC10595038

[11] Barkay O Bucksot L Sherman S . Endoscopic transpapillary gallbladder drainage with the SpyGlass cholangiopancreatoscopy system. Gastrointest Endosc. 2009;70(5):1039 1040. (10.1016/j.gie.2009.03.033) 19559418

[12] Shin JU Lee JK Kim KM . et al. Endoscopic naso-gallbladder drainage by using cholangioscopy for acute cholecystitis combined with cholangitis or choledocholithiasis (with video). Gastrointest Endosc. 2012;76(5):1052 1055. (10.1016/j.gie.2012.06.034) 23078929

[13] Kedia P Kuo V Tarnasky P . Digital cholangioscopy-assisted endoscopic gallbladder drainage. Gastrointest Endosc. 2017;85(1):257 258. (10.1016/j.gie.2016.07.016) 27449190

[14] Tyberg A Zerbo S Kahaleh M Sharaiha RZ . Digital cholangioscopy-assisted gallbladder drainage: seeing is accessing. Endoscopy. 2015;47(suppl 1 UCTN):E417. (10.1055/s-0034-1392655) 26397847

[15] Liu XG Huang XY Huang R Zhang RY Liu WH . Peroral cholangioscopy-guided transpapillary gallbladder drainage and cholecystolithotomy in the treatment of acute cholecystitis and cholelithiasis. Endoscopy. 2023;55(S 01):E686 E687. (10.1055/a-2067-4636) 37142247 PMC10159781

[16] Ridtitid W Piyachaturawat P Teeratorn N Angsuwatcharakon P Kongkam P Rerknimitr R . Single-operator peroral cholangioscopy cystic duct cannulation for transpapillary gallbladder stent placement in patients with acute cholecystitis at moderate to high surgical risk (with videos). Gastrointest Endosc. 2020;92(3):634 644. (10.1016/j.gie.2020.03.3866) 32330504

[17] Yoshida M Naitoh I Hayashi K , et al. Four-step classification of endoscopic transpapillary gallbladder drainage and the practical efficacy of cholangioscopic assistance. Gut Liver. 2021;15(3):476 485. (10.5009/gnl20238) 33402544 PMC8129659

[18] Zhou L Shen Y Xu B , et al. Feasibility of gallbladder lesion visualization using a novel ultrafine peroral cholangioscopy: a preliminary investigation. Dig Liver Dis. 2024;56(5):841 846. (10.1016/j.dld.2023.11.016) 38008699

[19] Chang AT Huang WH . Cholangioscopy complicated by gallbladder perforation. Gastrointest Endosc. 2019;89(5):1064 1065. (10.1016/j.gie.2019.01.007) 30639541

[20] Kaneko J Watahiki M Jindo O , et al. Gallbladder perforation following peroral cholangioscopy-guided lithotripsy: a case report. DEN Open. 2023;3(1):e237. (10.1002/deo2.237) 37091282 PMC10117168

[21] Kawai H Sato T Natsui M , et al. Mirizzi syndrome Type IV successfully treated with peroral single-operator cholangioscopy-guided electrohydraulic lithotripsy: a case report with literature review. Intern Med. 2022;61(23):3513 3519. (10.2169/internalmedicine.9526-22) 35569988 PMC9790796

[22] Li J Guo SJ Zhang JC , et al. Novel peroral cholangioscopy-directed lithotripsy using an ultraslim upper endoscope for refractory Mirizzi syndrome: a case report. Med (Baltim). 2020;99(45):e22649. (10.1097/MD.0000000000022649) PMC7647584 33157920

[23] Tsuyuguchi T Sakai Y Sugiyama H Ishihara T Yokosuka O . Long-term follow-up after peroral cholangioscopy-directed lithotripsy in patients with difficult bile duct stones, including Mirizzi syndrome: an analysis of risk factors predicting stone recurrence. Surg Endosc. 2011;25(7):2179 2185. (10.1007/s00464-010-1520-1) 21184106

[24] Bhandari S Bathini R Sharma A Maydeo A . Usefulness of single-operator cholangioscopy-guided laser lithotripsy in patients with Mirizzi syndrome and cystic duct stones: experience at a tertiary care center. Gastrointest Endosc. 2016;84(1):56 61. (10.1016/j.gie.2015.12.025) 26764195

[25] Kamada H Kobara H Yamana H , et al. Endoscopic direct visualization of gallbladder polypoid lesion using peroral digital single-operator cholangioscopy. Endoscopy. 2021;53(7):E263 E264. (10.1055/a-1252-2704) 33003215

[26] Irani SS Sharzehi K Siddiqui UD . AGA clinical practice update on role of EUS-guided gallbladder drainage in acute cholecystitis: commentary. Clin Gastroenterol Hepatol. 2023;21(5):1141 1147. (10.1016/j.cgh.2022.12.039) 36967319

[27] Peñas-Herrero I de la Serna-Higuera C Perez-Miranda M . Endoscopic ultrasound-guided gallbladder drainage for the management of acute cholecystitis (with video). J Hepatobil Pancreat Sci. 2015;22(1):35 43. (10.1002/jhbp.182) 25392972

[28] Anderloni A Buda A Vieceli F Khashab MA Hassan C Repici A . Endoscopic ultrasound-guided transmural stenting for gallbladder drainage in high-risk patients with acute cholecystitis: a systematic review and pooled analysis. Surg Endosc. 2016;30(12):5200 5208. (10.1007/s00464-016-4894-x) 27059975

[29] Song TJ Park DH Eum JB , et al. EUS-guided cholecystoenterostomy with single-step placement of a 7F double-pigtail plastic stent in patients who are unsuitable for cholecystectomy: a pilot study (with video). Gastrointest Endosc. 2010;71(3):634 640. (10.1016/j.gie.2009.11.024) 20189528

[30] Kamata K Takenaka M Kitano M , et al. Endoscopic ultrasound-guided gallbladder drainage for acute cholecystitis: long-term outcomes after removal of a self-expandable metal stent. World J Gastroenterol. 2017;23(4):661 667. (10.3748/wjg.v23.i4.661) 28216973 PMC5292340

[31] Widmer J Alvarez P Gaidhane M , et al. Endoscopic ultrasonography-guided cholecystogastrostomy in patients with unresectable pancreatic cancer using anti-migratory metal stents: a new approach. Dig Endosc. 2014;26(4):599 602. (10.1111/den.12163) 24102709

[32] Salameh H DiMaio CJ . Endoscopic retrograde cholangiopancreatography and endoscopic ultrasound-guided gallbladder drainage. Gastrointest Endosc Clin N Am. 2019;29(2):293 310. (10.1016/j.giec.2018.12.002) 30846154

[33] Teoh AYB Leung CH Tam PTH , et al. EUS-guided gallbladder drainage versus laparoscopic cholecystectomy for acute cholecystitis: a propensity score analysis with 1-year follow-up data. Gastrointest Endosc. 2021;93(3):577 583. (10.1016/j.gie.2020.06.066) 32615177

[34] Teoh AY Binmoeller KF Lau JY . Single-step EUS-guided puncture and delivery of a lumen-apposing stent for gallbladder drainage using a novel cautery-tipped stent delivery system. Gastrointest Endosc. 2014;80(6):1171. (10.1016/j.gie.2014.03.038) 24830582

[35] Mangiavillano B Moon JH Crinò SF , et al. Safety and efficacy of a novel electrocautery-enhanced lumen-apposing metal stent in interventional EUS procedures (with video). Gastrointest Endosc. 2022;95(1):115 122. (10.1016/j.gie.2021.07.021) 34339667

[36] Brandaleone L Franchellucci G Facciorusso A , et al. The use of a new dedicated electrocautery lumen-apposing metal stent for gallbladder drainage in patients with acute cholecystitis. Diagnostics (Basel). 2023;13(21). (10.3390/diagnostics13213341) PMC10650170 37958236

[37] Kamal F Khan MA Lee-Smith W , et al. Efficacy and safety of EUS-guided gallbladder drainage for rescue treatment of malignant biliary obstruction: a systematic review and meta-analysis. Endosc Ultrasound. 2023;12(1):8 15. (10.4103/EUS-D-21-00206) 36861505 PMC10134926

[38] Boregowda U Chen M Saligram S . Endoscopic Ultrasound-Guided Gallbladder Drainage versus Percutaneous gallbladder Drainage for Acute Cholecystitis: a Systematic Review and Meta-Analysis. Diagnostics (Basel). 2023;13(4). (10.3390/diagnostics13040657) PMC9954901 36832143

[39] Fabbri C Binda C Sbrancia M , et al. Determinants of outcomes of transmural EUS-guided gallbladder drainage: systematic review with proportion meta-analysis and meta-regression. Surg Endosc. 2022;36(11):7974 7985. (10.1007/s00464-022-09339-y) 35652964

[40] Ahmed O Rogers AC Bolger JC , et al. Meta-analysis of outcomes of endoscopic ultrasound-guided gallbladder drainage versus percutaneous cholecystostomy for the management of acute cholecystitis. Surg Endosc. 2018;32(4):1627 1635. (10.1007/s00464-018-6041-3) 29404731

[41] Podboy A Yuan J Stave CD Chan SM Hwang JH Teoh AYB . Comparison of EUS-guided endoscopic transpapillary and percutaneous gallbladder drainage for acute cholecystitis: a systematic review with network meta-analysis. Gastrointest Endosc. 2021;93(4):797 804.e1. (10.1016/j.gie.2020.09.040) 32987004

[42] Chan SM Teoh AYB Yip HC Wong VWY Chiu PWY Ng EKW . Feasibility of per-oral cholecystoscopy and advanced gallbladder interventions after EUS-guided gallbladder stenting (with video). Gastrointest Endosc. 2017;85(6):1225 1232. (10.1016/j.gie.2016.10.014) 27756612

[43] Choi JH Lee SS Choi JH , et al. Long-term outcomes after endoscopic ultrasonography-guided gallbladder drainage for acute cholecystitis. Endoscopy. 2014;46(8):656 661. (10.1055/s-0034-1365720) 24977397

[44] Ge N Sun S Sun S Wang S Liu X Wang G . Endoscopic ultrasound-assisted transmural cholecystoduodenostomy or cholecystogastrostomy as a bridge for per-oral cholecystoscopy therapy using double-flanged fully covered metal stent. BMC Gastroenterol. 2016;16:9. (10.1186/s12876-016-0420-9) 26782105 PMC4717638

[45] Liu H Lu Y Shen K Zhou M Mao X Li R . Advances in the management of gallbladder polyps: establishment of predictive models and the rise of gallbladder-preserving polypectomy procedures. BMC Gastroenterol. 2024;24(1):7. (10.1186/s12876-023-03094-7) 38166603 PMC10759486

[46] Sugiyama M Atomi Y Yamato T . Endoscopic ultrasonography for differential diagnosis of polypoid gall bladder lesions: analysis in surgical and follow up series. Gut. 2000;46(2):250 254. (10.1136/gut.46.2.250) 10644321 PMC1727801

[47] Kim SY Cho JH Kim EJ , et al. The efficacy of real-time colour Doppler flow imaging on endoscopic ultrasonography for differential diagnosis between neoplastic and non-neoplastic gallbladder polyps. Eur Radiol. 2018;28(5):1994 2002. (10.1007/s00330-017-5175-3) 29218621

[48] Singla V Agarwal R Anikhindi SA , et al. Role of EUS-FNA for gallbladder mass lesions with biliary obstruction: a large single-center experience. Endosc Int Open. 2019;7(11):E1403 E1409. (10.1055/a-0982-2862) 31673611 PMC6805207

[49] Kang SH Joo JS Kim SH Kim KH Lee ES . EUS-guided fine-needle biopsy of gallbladder polypoid lesions [video]. VideoGIE. 2020;5(4):151 153. (10.1016/j.vgie.2019.12.004) 32258846 PMC7125392

[50] Shen Y Cao J Zhou X , et al. Endoscopic ultrasound-guided cholecystostomy for resection of gallbladder polyps with lumen-apposing metal stent. Med (Baltim). 2020;99(43):e22903. (10.1097/MD.0000000000022903) PMC7581112 33120842

[51] Tsin DA Sequeria RJ Giannikas G . Culdolaparoscopic cholecystectomy during vaginal hysterectomy. JSLS. 2003;7(2):171 172. 12856851 PMC3015482

[52] Dhillon KS Awasthi D Dhillon AS . Natural orifice transluminal endoscopic surgery (hybrid) cholecystectomy: the Dhillon technique. J Minim Access Surg. 2017;13(3):176 181. (10.4103/0972-9941.207838) 28607283 PMC5485805

[53] Zornig C Siemssen L Emmermann A , et al. NOTES cholecystectomy: matched-pair analysis comparing the transvaginal hybrid and conventional laparoscopic techniques in a series of 216 patients. Surg Endosc. 2011;25(6):1822 1826. (10.1007/s00464-010-1473-4) 21181204

[54] Zornig C Mofid H Emmermann A Alm M von Waldenfels HA Felixmüller C . Scarless cholecystectomy with combined transvaginal and transumbilical approach in a series of 20 patients. Surg Endosc. 2008;22(6):1427 1429. (10.1007/s00464-008-9891-2) 18398645

[55] Bulian DR Knuth J Cerasani N Sauerwald A Lefering R Heiss MM . Transvaginal/transumbilical hybrid--NOTES--versus 3-trocar needlescopic cholecystectomy: short-term results of a randomized clinical trial. Ann Surg. 2015;261(3):451 458. (10.1097/SLA.0000000000000218) 24108196 PMC4337615

[56] Brescia A Masoni L Gasparrini M , et al. Laparoscopic assisted transvaginal cholecystectomy: single centre preliminary experience. Surgeon. 2013;11(suppl 1):S1 S5. (10.1016/j.surge.2012.09.003) 23182808

[57] Noguera JF Cuadrado A Dolz C Olea JM García JC . Prospective randomized clinical trial comparing laparoscopic cholecystectomy and hybrid natural orifice transluminal endoscopic surgery (NOTES) (NCT00835250). Surg Endosc. 2012;26(12):3435 3441. (10.1007/s00464-012-2359-4) 22648123

[58] Borchert DH Federlein M Fritze-Büttner F , et al. Postoperative pain after transvaginal cholecystectomy: single-center, double-blind, randomized controlled trial. Surg Endosc. 2014;28(6):1886 1894. (10.1007/s00464-013-3409-2) 24464385

[59] Peng C Ling Y Ma C , et al. Safety outcomes of NOTES cholecystectomy versus laparoscopic cholecystectomy: a systematic review and meta-analysis. Surg Laparosc Endosc Percutan Tech. 2016;26(5):347 353. (10.1097/SLE.0000000000000284) 27557339 PMC5054957

[60] Wood SG Solomon D Panait L Bell RL Duffy AJ Roberts KE . Transvaginal cholecystectomy: effect on quality of life and female sexual function. JAMA Surg. 2013;148(5):435 438. (10.1001/jamasurg.2013.108) 23677408

[61] Shang L Shen X Niu W , et al. Update on the natural orifice transluminal endoscopic surgery for gallbladder preserving gallstones therapy: a review. Med (Baltim). 2022;101(46):e31810. (10.1097/MD.0000000000031810) PMC9678607 36401453

[62] Liu B Du B Pan Y . Video of the month: transrectal gallbladder-preserving cholecystolithotomy via pure natural orifice transluminal endoscopic surgery: first time in humans. Am J Gastroenterol. 2015;110(12):1655. (10.1038/ajg.2015.266) 26673494

[63] Li Y Han S . Transgastric endoscopic gallbladder polypectomy and cholecystolithiasis: a case report. Exp Ther Med. 2020;19(1):95 98. (10.3892/etm.2019.8195) 31853277 PMC6909710

[64] Zhang Y Mao XL Zhou XB , et al. Feasibility of transgastric endoscopic gallbladder-preserving surgery for benign gallbladder diseases (with video). Surg Endosc. 2022;36(4):2705 2711. (10.1007/s00464-021-08890-4) 35075524

[65] Ullah S Yang BH Liu D , et al. Are laparoscopic cholecystectomy and natural orifice transluminal endoscopic surgery gallbladder preserving cholecystolithotomy truly comparable? a propensity matched study. World J Gastrointest Surg. 2022;14(5):470 481. (10.4240/wjgs.v14.i5.470) 35734621 PMC9160690

